# The Impact of Accidental Hypothermia on Mortality in Trauma Patients Overall and Patients with Traumatic Brain Injury Specifically: A Systematic Review and Meta-Analysis

**DOI:** 10.1007/s00268-020-05750-5

**Published:** 2020-08-28

**Authors:** David Rösli, Beat Schnüriger, Daniel Candinas, Tobias Haltmeier

**Affiliations:** Department of Visceral Surgery and Medicine, Inselspital, Bern University Hospital, University of Bern, Bern, Switzerland

## Abstract

**Background:**

Accidental hypothermia is a known predictor for worse outcomes in trauma patients, but has not been comprehensively assessed in a meta-analysis so far. The aim of this systematic review and meta-analysis was to investigate the impact of accidental hypothermia on mortality in trauma patients overall and patients with traumatic brain injury (TBI) specifically.

**Methods:**

This is a systematic review and meta-analysis using the Ovid Medline/PubMed database. Scientific articles reporting accidental hypothermia and its impact on outcomes in trauma patients were included in qualitative synthesis. Studies that compared the effect of hypothermia vs. normothermia at hospital admission on in-hospital mortality were included in two meta-analyses on (1) trauma patients overall and (2) patients with TBI specifically. Meta-analysis was performed using a Mantel–Haenszel random-effects model.

**Results:**

Literature search revealed 264 articles. Of these, 14 studies published 1987–2018 were included in the qualitative synthesis. Seven studies qualified for meta-analysis on trauma patients overall and three studies for meta-analysis on patients with TBI specifically. Accidental hypothermia at admission was associated with significantly higher mortality both in trauma patients overall (OR 5.18 [95% CI 2.61–10.28]) and patients with TBI specifically (OR 2.38 [95% CI 1.53–3.69]).

**Conclusions:**

In the current meta-analysis, accidental hypothermia was strongly associated with higher in-hospital mortality both in trauma patients overall and patients with TBI specifically. These findings underscore the importance of measures to avoid accidental hypothermia in the prehospital care of trauma patients.

**Electronic supplementary material:**

The online version of this article (10.1007/s00268-020-05750-5) contains supplementary material, which is available to authorized users.

## Background

Accidental hypothermia occurs frequently in trauma patients. Up to 66% of patients being admitted to the emergency department (ED) for major trauma have been reported to suffer from accidental hypothermia [1]. Hypothermia is commonly defined as a core body temperature < 35 °C and further classified into mild (35 °C–32 °C), moderate (32 °C–28 °C), and severe (< 28 °C) [2]. The etiology is multifactorial, comprising environmental exposure, hemorrhagic shock, not sufficiently prewarmed solutions for volume resuscitation and drugs causing reduced vasoconstriction and a higher shivering threshold, such as anesthetics and muscle relaxants [3–5].

Accidental hypothermia has been described as a predictor for worse outcomes, including higher mortality, higher blood product transfusion requirements, and longer intensive care unit (ICU) and hospital length of stay (LOS), both in trauma patients overall and patients with traumatic brain injury (TBI) specifically [6–10]. In severely injured trauma patients, mortality increases with the degree of hypothermia [9].

On the other hand, potential beneficial effects of hypothermia have been discussed in trauma patients, too [11–13]. In analogy to the targeted temperature management in patients with cardiac arrest, the rationale for induced hypothermia in trauma patients with TBI is to decrease the deleterious effects of secondary brain injury. Hypothermia is thought to reduce the effects of secondary brain injury through multiple mechanisms, including reduced excitotoxicity, oxidative stress, apoptosis, autophagy, and inflammation [14, 15]. Experimental studies suggest that mild hypothermia may alter the inflammatory response after TBI [16, 17]. Furthermore, animal models revealed reduced mortality, an improved behavioral outcome, and a diminished disruption of the blood–brain barrier in animals subjected to mild therapeutic hypothermia after having suffered from TBI [18, 19].

Although accidental hypothermia is important not only in the clinical management of trauma patients, but also in traumatic brain injury research, it has not yet been comprehensively addressed in a systematic literature review and meta-analysis. The aim of this literature review and meta-analysis was to assess the effect of accidental hypothermia on outcomes in trauma patients overall and patients with TBI specifically. We hypothesized that accidental hypothermia has a strong adverse impact on outcomes both in trauma patients overall and patients with TBI specifically.

## Methods

This is a systematic literature review and meta-analysis exploring the impact of accidental hypothermia on outcomes in trauma patients overall and patients with TBI specifically.

PRISMA 2009 Guidelines [20, 21] were followed throughout the systematic literature review, reporting of the data, and discussion (Supplemental Table 1).

Articles were assessed, and data were extracted by two reviewers (DR, TH). Differences were resolved by consensus.

This study has been registered at ClinicalTrials.gov (ID NCT04332237).

### Literature search

A systematic literature search was performed using *Ovid Medline* [22] and *PubMed* [23] (Medline database, US National Library of Medicine). The search strategy was based on the PICOS strategy [24, 25]. Medical Subject Headings (MeSH) were used as search terms whenever feasible [26]. No additional filters were utilized. Literature search was conducted in August 2019.

Literature search was performed including the following search terms:

(multiple trauma OR polytrauma OR major trauma OR ISS OR APACHE) AND (hypothermia OR accidental hypothermia) AND (mortality OR intensive care unit OR survival rate OR length of stay OR outcome OR bleeding OR ventilator).

All abstracts were screened for eligibility. Articles were included in the qualitative synthesis if they met the following criteria:

1.: Study in trauma patients.
2.: Reported body temperature and time of measurement.
3.: Reported impact of accidental hypothermia on outcomes.

References relevant to the topic listed in the bibliography of articles found through the search strategy mentioned above were also included. Only original research articles in English language were considered for inclusion. Articles with no relevance to the topic, case reports, systematic reviews, and articles covering therapeutic hypothermia were excluded. Full text articles of abstracts that met the inclusion criteria were subsequently assessed.

If two or more studies acquired data from the same database during an overlapping time period and included the same patient population, the study covering the longest time period or study that qualified for meta-analysis was selected for inclusion. The other studies were excluded from the systematic review and meta-analysis.

### Endpoint

The endpoint investigated in the current systematic review and meta-analysis was in-hospital mortality. In studies that reported overall mortality or mortality not further specified, the reported mortality rate was assumed to correspond to in-hospital mortality.

### Meta-analysis

Two meta-analyses were preformed including (1) trauma patients overall and (2) patients with TBI specifically. Studies that reported in-hospital mortality of hypothermic and normothermic trauma patients overall or patients with TBI specifically, based on the temperature measured at ED admission, were included in meta-analyses. The number of survivors and non-survivors in the hypothermic and normothermic group was extracted from these studies. For studies that reported mortality of hypothermic and normothermic patients as percentages only, the actual number of patients was calculated from the percentages.

Meta-analysis was conducted using a Mantel–Haenszel random-effects model. The estimated effect size for mortality was reported as odds ratio (OR) and 95% confidence interval (CI) for each study as well as for the overall cohort. Statistical heterogeneity of the studies included was measured using Cochrane Q statistics and I-square [27, 28] and interpreted based on the current consensus-based recommendations by Gagnier et al. [29].

Sensitivity analysis was performed by repeating the Mantel–Haenszel random-effects model in the subgroups of studies using the same definition of hypothermia, studies with similar inclusion criteria regarding the ISS, studies published during the last 10 years, and studies including more than 10,000 patients.

Statistical analysis was performed using Review Manager (RevMan) Version 5.3. (Copenhagen: The Nordic Cochrane Collaboration, 2014).

### Quality assessment

The quality of the studies incorporated in the systematic literature review and meta-analysis was assessed using the Newcastle–Ottawa Scale (NOS) for cohort studies [30] (Supplemental Table 2).

## Results

### Study selection

**Fig. 1:**
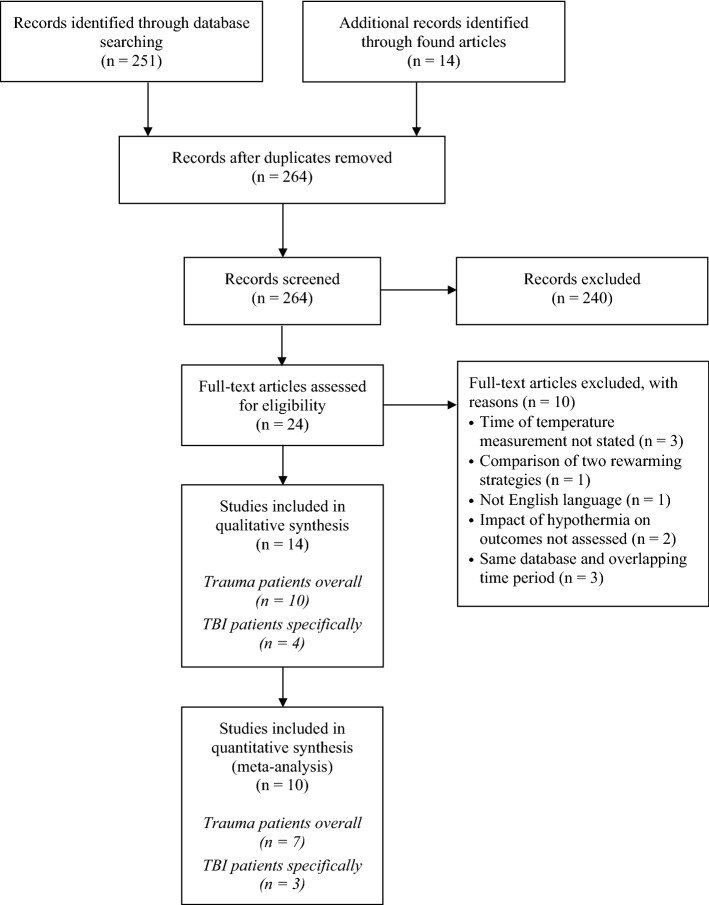
PRISMA 2009 flow diagram

A total of 251 articles were obtained from Medline. Similar search results were obtained using PubMed. Fourteen additional publications cited in these 251 articles were found to be relevant to the topic, too. After removal of duplicates, abstracts of 264 articles were screened for eligibility. Abstracts of twenty-four studies met the inclusion criteria. Of these, the full text articles were assessed (Fig. 1).

Ten studies reported data from the same database or registry during an overlapping time period [6–8, 10, 31–36]. Of these, three studies were excluded from the review and meta-analysis [10, 33, 34]. Three other studies included patients suffering from TBI specifically. These three studies were excluded from meta-analysis for trauma patients overall, but included in meta-analysis for patients with TBI specifically [6, 7, 36].

Altogether, after the assessment of the full text articles, a total of ten studies were excluded and 14 studies were included in qualitative synthesis.

### Study characteristics

Study characteristics are outlined in Table 1. Publication years ranged from 1987 to 2018. Eleven studies were retrospective analyses [6–8, 31, 32, 35–40], and three studies were designed prospectively [1, 41, 42].

**Table 1:** Study characteristics

Author, *Journal,* Year	Study type	Study size (*n* =)	Definition of hypothermia	Time of temperature measurement	Normothermic at admission *n* (%)	Hypothermic at admission *n* (%)
*Studies including trauma patients overall*						
Aitken et al., *Resuscitation*, 2009	Retrospective database analysis	2,026	< 35 °C	ED admission	1,902 (93.9)	124 (6.1)
Arthurs et al., *Am J Surg*, 2006	Retrospective single center	2,848	< 36 °C	ED admission	2,331 (81.8)	517 (18.2)
Balvers et al., *J Emerg Trauma Shock*, 2016	Retrospective multicenter	953	≤ 35 °C	ICU admission	599 (62.9)	354 (37.1)
Bukur et al., *J Trauma Acute Care Surg*, 2012	Retrospective database analysis	21,023	≤ 36.5 °C	ED admission	11,642 (55.4)	9,381 (44.6)
Hsieh et al., *Int J Env Res Pub He*, 2018	Retrospective single center	13,769	< 36 °C	ED admission	13,368 (97.1)	401 (2.9)
Ireland et al., *Resuscitation*, 2011	Prospective single center	732	< 35 °C	ED admission	635 (86.7)	97 (13.3)
Luna et al., *J Trauma*, 1987	Prospective single center	94	≤ 36 °C	Field, ED, OR	32 (34)	62 (66)
Martin et al., *Shock*, 2005	Retrospective database analysis	700,304	< 35 °C	ED admission	689,278 (98.4)	11,026 (1.6)
Mommsen et al., *Injury*, 2013	Retrospective single center	310	< 35 °C	ED admission	196 (63.2)	114 (36.8)
Wang et al., *Crit Care Med*, 2005	Retrospective database analysis	38,520	≤ 35 °C	ED admission	36,599 (95)	1,921 (5)
*Studies including patients with traumatic brain injury*						
Bukur et al., *J Surg Res*, 2012	Retrospective database analysis	1,834	≤ 35 °C	ED admission	1,790 (97.6)	44 (2.4)
Rubiano et al., *Injury*, 2013	Retrospective database analysis	9,094^+^	≤ 35 °C	ED admission	7,431 (67.4)	1,663 (32.6)
Tohme et al., *Scand J Trauma Resusc Emerg Med*, 2014	Prospective multicenter cohort	589	≤ 35 °C	ED admission	443 (75.2)	146 (24.8)
Winkelmann et al., *Eur J Trauma Emerg Surg*, 2018	Retrospective single center	278	< 35 °C	ED admission	176 (63.3)	102 (36.7)

^+^Excluding 1,939 patients with no temperature recorded

### Traumatic brain injury

Four studies included patients with severe traumatic brain injury and accidental hypothermia specifically. The study by Bukur et al. included patients with isolated TBI [36], while the other three studies by Rubiano et al., Tohme et al., and Winkelmann et al. were comprised of patients suffering from concomitant TBI, i.e., patients with extracranial injuries were not excluded [6, 7, 42].

### Definition of hypothermia and temperature measurement

Definitions of hypothermia used in the studies included are outlined in Table 1. Temperature measurement methods were specified in six studies and comprised nasopharyngeal [39], esophageal [1, 7, 31, 32, 41], bladder [7, 31, 32], rectal [32, 39], tympanic [32, 41], axillary [32], and oral [32] temperature probes. Eight studies did not describe the exact method of temperature measurement [6, 8, 35–38, 40, 42].

### Patient characteristics

Patient characteristics are summarized in Table 2. Included patients were predominantly male (range 71–79%) [6, 31, 35, 36, 39, 41, 42]. Eight studies compared the age of the normothermic and hypothermic patient groups [7, 8, 31, 35–37, 39, 40]. In the studies by Bukur et al. and Martin et al., hypothermic patients were significantly older than normothermic patients [8, 35]. In the other six studies, no significant association of age and hypothermia was found [7, 31, 36, 37, 39, 40].

**Table 2:** Patient characteristics

Author, *Journal,* Year	Age overall (years)	Male sex (%)	Age normothermic (years)	Age hypothermic (years)	*p*-Value (age)	ISS overall	ISS normothermic	ISS hypothermic	*p*-Value (ISS)
*Studies including trauma patients overall*									
Aitken et al., *Resuscitation*, 2009	–	–	39 (0–101)^†^	41 (0–96)^†^	0.73	–	–	–	N/A
Arthurs et al., *Am J Surg*, 2006	–	–	–	–	N/A	–	11.2 (9)^+^	–	N/A
Balvers et al., *J Emerg Trauma Shock*, 2016	–	79%	44 (29–60)^‡^	46 (30–65)^‡^	0.242	–	24 (12)^+^	28 (14)^+^	< 0.001
Bukur et al., *J Trauma Acute Care Surg*, 2012	39.9 (19.5)^+^	73%	38.1 (18.7)^+^	42.2 (20.3)^+^	< 0.001	12.0 (9.9)^+^	10.9 (9.3)^+^	13.4 (10.5)^+^	< 0.001
Hsieh et al., *Int J Env Res Pub He*, 2018	–	–	54.3 (19.4)^+^	53.3 (19.0)^+^	0.293	–	9 (4–10)^‡^	10 (5–20)^‡^	< 0.001
Ireland et al., *Resuscitation*, 2011	45.8 (20.6)^+^	76%	–	–	N/A	22 (17–29)^‡^	–	–	N/A
Luna et al., *J Trauma*, 1987	–	–	35 (14–83)^#^	–	N/A	–	28 (4–50)^#^	–	N/A
Martin et al., *Shock*, 2005	–	–	37.8 (22.9)^+^	39.4 (22.4)^+^	< 0.001	–	8.9 (8.4)^+^	20.4 (15.5)^+^	< 0.001
Mommsen et al., *Injury*, 2013	41.9 (17.5)^+^	71%	41.1 (16.5)^+^	42.8 (19.1)^+^	0.483	29.7 (10.2)^+^	28.8 (9.8)^+^	31.2 (10.7)^+^	0.048
Wang et al., *Crit Care Med*, 2005	–	–	–	–	N/A	–	–	–	N/A
*Studies including patients with traumatic brain injury*									
Bukur et al., *J Surg Res*, 2012	39.4 (24.4)^+^	76%	39.4 (24.4)^+^	41.6 (25.7)^+^	0.374	17.2 (6.5)^+^	17.2 (6.5)^+^	20.7 (7.3)^+^	< 0.001
Rubiano et al., *Injury*, 2013	40.1 (35)^+^	72%	–	–	N/A		–	–	N/A
Tohme et al., *Scand J Trauma Resusc Emerg Med*, 2014	55 (33–70)^‡^	75%	–	–	N/A	25 (21–34)^‡^	–	–	N/A
Winkelmann et al., *Eur J Trauma Emerg Surg*, 2018	42.6 (19.4)^+^	–	44.5 (20.2)^+^	39.3 (17.6)^+^	0.054	32.8 (10.7)^+^	31.2 (10.1)^+^	35.6 (11.1)^+^	0.001

^+^Mean (SD), ^†^median (range), ^‡^median (IQR), ^#^mean (range), N/A: not applicable

Eight studies assessed the injury severity in normothermic and hypothermic patients separately. In seven studies, a significantly higher ISS was found in hypothermic compared to normothermic patients [7, 8, 31, 35, 36, 39, 40]. In the study by Wang et al., the proportion of patients with higher New Injury Severity Scores (NISS) was higher in the hypothermic compared to the normothermic group [32].

In the twelve studies that reported temperature at ED admission, the proportion of hypothermic patients ranged from 1.6 to 44.6% [6–8, 31, 32, 35–38, 40–42].

### Mortality

Four studies specifically reported in-hospital mortality [32, 35, 37, 40]. Balvers et al. reported both the 24-h and 28-day mortality rate [39]. Tohme et al. described 14-day mortality [42]. Eight studies reported overall mortality or mortality without further specification [1, 6–8, 31, 36, 38, 41].

In seven studies, death rates were significantly higher in hypothermic trauma patients compared to normothermic patients [8, 31, 35, 37, 39–41]. Bukur et al., Rubiano et al., Tohme et al., and Winkelmann et al., in their cohorts consisting of patients with TBI specifically, observed a significantly higher mortality rate when accidental hypothermia was present at hospital admission [6, 7, 36, 42].

In thirteen studies, the effect of hypothermia on mortality was adjusted for various covariates, including the injury severity. Eleven studies adjusted for the ISS [7, 8, 31, 35–42] and two studies for the NISS [6, 32] in multivariable analysis for mortality. In eleven studies, hypothermia was reported as an independent predictor for mortality, even after adjustment for the injury severity and other covariates [6, 8, 32, 35–42]. In two studies, the effect of hypothermia on mortality did not remain significant after adjustment in multivariable analysis [7, 31] (Table 3).

**Table 3:** Mortality, blood product transfusion requirements, and coagulation

Author,* Journal*, Year	Mortality normothermic *n* (%)	Mortality hypothermic *n* (%)	*p*-Value (mortality)	Adjusted effect of hypothermia on mortality^*^ OR (95% CI)	PRBC (normothermia vs. hypothermia) (units)	FFP (normothermia vs. hypothermia) (units)	PLT (normothermia vs. hypothermia) (units)	INR (normothermia vs. hypothermia)
*Studies including trauma patients overall*								
Aitken et al., *Resuscitation*, 2009	217 (11.4)	50 (40.3)	< 0.001	4.05 (2.26–7.24)	–	–	–	–
Arthurs et al., *Am J Surg*, 2006	–	–	–	3.8 (2.1–6.9)	4.8 (5)^+^ (normothermia), 6.5 (5)^+^ (34–36 °C) 9.6 (9)^+^ (< 34 °C) p < 0.01^†^	4.9 (5)^+^ (normothermia), 5.5 (4)^+^ (34–36 °C) 6.4 (4)^+^ (< 34 °C) p = 0.214^†^	–	–
Balvers et al., *J Emerg Trauma Shock*, 2016	11 (2)^a^ 57 (10)^b^	29 (8)^a^ 101 (29)^b^	< 0.001 < 0.001	2.72 (1.18 – 6.29)^a^ 2.82 (1.83–4.35)^b^	–	–	–	1.14 (1.0–1.4), 1.24 (1.1–1.6)^‡^
Bukur et al., *J Trauma Acute Care Surg*, 2012	396^#^ (3.4)	516^#^ (5.5)	< 0.001	2.0 (1.5–2.6)^d^	–	–	–	–
Hsieh et al., *Int J Env Res Pub He*, 2018	307 (2.3)	54 (13.5)	< 0.001	0.66 (0.54 – 0.80)^e^	–	–	–	–
Ireland et al., *Resuscitation*, 2011	38 (6.0)	29 (29.9)	< 0.001	3.44 (1.48–7.99)	4 (2–6), 4 (3–9)^‡^ p = 0.088	600 (600 – 1,500)^‡**^, 1,500 (600 – 1,800)^‡**^ p = 0.135	4 (2–5), 2 (1–5)^‡^ p = 0.531	1.0 (1.0–1.1), 1.2 (1.0–1.6)^‡^
Luna et al., *J Trauma*, 1987	−(22)	–	–	–	–	–	–	–
Martin et al., *Shock*, 2005	20,678^#^ (3.0)	2,812^#^ (25.5)	< 0.001	1.54 (1.4–1.71)	–	–	–	–
Mommsen et al., *Injury*, 2013	11 (5.6)	16 (14)	0.02	2.05 (0.86–4.92)	11.5 (14.4), 18.2 (19.2)^+^ p = 0.005	7.6 (11.5), 12.5 (14.1)^+^ p < 0.001	1.1 (3.6), 2.1 (3.4)^+^ p < 0.001	–
Wang et al., *Crit Care Med*, 2005	1,616 (4.4)	511 (26.6)	–	3.03 (2.62–3.51)	–	–	–	–
*Studies including patients with traumatic brain injury*								
Bukur et al., *J Surg Res*, 2012	125^#^ (7.0)	11^#^ (25.0)	< 0.001	2.5 (1.1–6.3)	–	–	–	–
Rubiano et al., *Injury*, 2013	2,781 (37.4)	896 (53.9)	< 0.001	1.70 (1.50–1.93)	–	–	–	–
Tohme et al., *Scand J Trauma Resusc Emerg Med*, 2014	103 (23.3)^c^	56 (38.4)^c^	< 0.001	1.42 (1.00–2.01)^c^	–	–	–	–
Winkelmann et al., *Eur J Trauma Emerg Surg*, 2018	23 (13.1)	24 (23.5)	0.03	–	7.6 (10.4), 14.0 (15.6)^+^ *p* < 0.001	5.0 (8.0), 9.9 (13.0)^+^ *p* < 0.001	0.6 (1.5), 1.5 (2.4)^+^ p < 0.001	–

*Adjusted in multivariable analysis, ^**^[ml], ^+^mean (SD), ^‡^median (IQR), ^†^significance between the three groups, ^#^calculated from percentage, ^a^24-h mortality, ^b^28-day mortality, ^c^14-day mortality, ^d^transfused patients, ^e^temperature maintained as continuous variable

### Meta-analysis for mortality

**Fig. 2:**
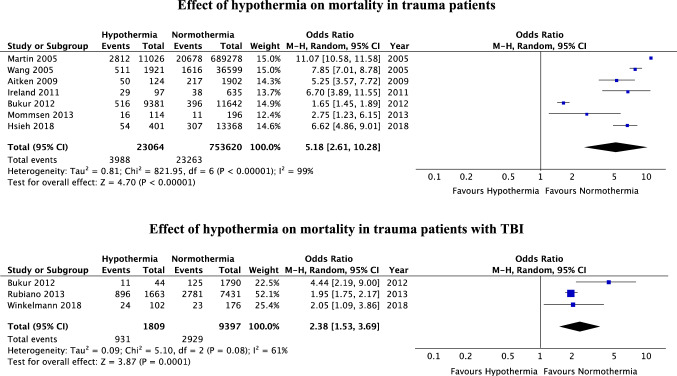
Meta-analysis

Seven studies comparing in-hospital mortality of normothermic and hypothermic trauma patients at ED admission were included in meta-analysis for trauma patients overall [8, 31, 32, 35, 37, 40, 41]. Meta-analysis for trauma patients overall revealed a significantly higher mortality in patients with hypothermia on admission (OR 5.18 [95% CI 2.61–10.28]) (Fig. 2).

Three studies were included in meta-analysis for patients with TBI specifically [6, 7, 36]. Meta-analysis for patients with TBI specifically revealed significantly higher mortality in hypothermic compared to normothermic patients (OR 2.38 [95% CI 1.53–3.69]) (Fig. 2).

In sensitivity analysis including studies with the same definition of hypothermia (temperature < 35 °C, four studies, 703,372 patients [8, 31, 37, 41]), studies with similar patient inclusion criteria regarding the ISS (ISS ≥ 16, three studies, 3,068 patients [31, 37, 41]), studies published during the last 10 years (four studies, 35,834 patients [31, 35, 40, 41]), and studies with more than 10,000 patients (four studies, 773,616 patients [8, 32, 35, 40]), hypothermia on admission was associated with significantly higher mortality, too (OR 6.15 [95%CI 3.43–11.05], *I*^2^ = 90%; OR 5.02 [95%CI 3.35–7.52], *I*^2^ = 39%; OR 3.76 [95%CI 1.48–9.52], *I*^2^ = 96%; and OR 5.55 [95%CI 2.23–3.84], *I*^2^ = 100%).

### Blood product transfusion and coagulation disorder

Transfused blood products in normothermic and hypothermic trauma victims were explored by four studies [7, 31, 38, 41]. In two studies, packed red blood cells (PRBCs) transfusion rates were significantly higher in hypothermic patients [7, 31]. In the other two studies, no significant difference in PRBC transfusions between hypothermic and normothermic patients was found [38, 41].

Mommsen et al. and Winkelmann et al. reported significantly higher fresh frozen plasma (FFP) and platelet (PLT) transfusion rates in hypothermic trauma patients [7, 31].

INR values were provided by Balvers et al. and Ireland et al. and were significantly higher in hypothermic individuals [39, 41] (Table 3).

### Quality assessment

Overall, the quality of the studies included was satisfactory. Twelve studies received nine out of nine stars, one study received eight stars, and one study seven stars (Supplemental Table 2).

## Discussion

The aim of this literature review and meta-analysis was to systematically assess the impact of accidental hypothermia on outcomes in trauma patients overall and in patients with TBI specifically. Meta-analysis revealed accidental hypothermia as a significant predictor for higher in-hospital mortality both in trauma patients overall and patients with TBI specifically. With an overall OR of 5.18 for trauma patients and 2.38 for patients with TBI specifically, hypothermia had a strong impact on mortality.

Taking into account the strong impact of accidental hypothermia on mortality, efforts should be made to prevent hypothermia in trauma patients whenever possible. Persistent hypothermia in severely injured trauma patients decreases the likelihood of successful resuscitation [43]. Consequently, it must be prevented in the prehospital setting, but also during early hospital care. Measures to avoid and treat hypothermia include the removal of wet clothing, a warm environment, warming blankets, warm parenteral fluids including blood products, avoidance of unnecessary anesthesia, avoidance of prolonged surgery with an open abdomen, warm thoracic or abdominal lavage, and, in cases of severe hypothermia with cardiac instability or arrest, rewarming with extracorporeal membrane oxygenation or cardiopulmonary bypass [2, 44–46]. These techniques to avoid hypothermia in trauma patients and combat casualties are part of the current Advanced Trauma Life Support [47] and Tactical Combat Casualty Care [48] guidelines.

Trauma-induced coagulopathy is frequent in severely injured patients and has been shown to be associated with higher mortality [49, 50]. Hypothermia, acidosis, and hemodilution due to volume resuscitation share complex interactions and influence each other disadvantageously, leading to an aggravation of coagulopathy in trauma patients [51, 52].

In the current review, two studies reported significantly higher transfusion requirements [7, 31] and two other studies significantly higher INR values [39, 41] in hypothermic patients compared to normothermic patients. In the setting of severely injured trauma patients, higher transfusion requirements and INR values may serve as surrogate markers for coagulopathy. Thus, the higher mortality rate in patients suffering from accidental hypothermia in the studies included in this review may be related to hypothermia-induced coagulopathy.

However, as in the studies included the effect of hypothermia on transfusion requirements and the INR was not adjusted for other clinically important factors, such as the injury severity and transport time, these results may be confounded.

In this systematic review, three studies that included patients with TBI specifically reported a higher mortality rate in hypothermic compared to normothermic patients [6, 36, 42]. Of note, all three studies investigated patients with severe TBI, but only one included patients with isolated severe TBI [36]. Any conclusion concerning the effect of hypothermia on mortality in patients suffering from isolated TBI and mild or moderate TBI is therefore limited.

Beneficial effects of hypothermia on secondary brain injury have previously been discussed in the literature [11–13]. Hypothermia leads to the suppression of multiple damaging mechanisms in the injured brain, including excitotoxicity, oxidative stress, apoptosis, autophagy, and inflammation [14, 15]. Furthermore, experimental studies suggest that hypothermia is associated with a decreased blood–brain barrier permeability and inhibition of diffuse axonal injury [18, 53].

However, these potential neuroprotective effects of hypothermia in traumatic brain injury are most likely outweighed by the deleterious effect of the trauma-induced coagulopathy and consecutive aggravated intracranial bleeding [11, 54].

Hypothermia due to hypothalamic dysfunction has been described in the literature [55] and may have potentially contributed to the higher mortality in hypothermic patients with severe TBI. However, hypothermia in patients with hypothalamic dysfunction would not be expected at ED admission, but rather later in the hospital course.

Further research on the effect of hypothermia in patients suffering from TBI, especially those with isolated brain injury, is warranted.

In the existing literature, two other meta-analyses by Harris et al. and McHugh et al. reported hypothermia in patients with traumatic brain injury [56, 57]. However, the effect of accidental hypothermia at ED admission was not addressed in these studies, and none of the studies included in these meta-analyses met the inclusion criteria for the current analysis. Harris et al. included seven randomized controlled trials comparing the effect of induced hypothermia versus normothermia in patients with posttraumatic head injury on several endpoints, including the Glasgow Outcome Scale Score, intracranial pressure, pneumonia, cardiac arrhythmia, prothrombin time, and partial thromboplastin time. No benefit of therapeutic hypothermia on these endpoints was shown [57]. The meta-analysis by McHugh et al. [56] incorporated ten studies from the International Mission for Prognosis And Clinical Trial (IMPACT) database [58]. The main outcome was the Glasgow Outcome Scale Score at 6 months in patients having suffered from moderate or severe TBI. In this meta-analysis, hypothermia was associated with adverse outcomes, too [56].

This systematic review and meta-analysis has several limitations.

First, eleven studies were designed retrospectively [6–8, 31, 32, 35–40], and only three studies prospectively [1, 41, 42]. Thus, most studies included had the known limitations of retrospective analyses.

Second, substantial statistical heterogeneity was found both in meta-analysis on trauma patients overall and patients with TBI specifically. Statistical heterogeneity of the current meta-analysis may be explained by methodological and clinical heterogeneity of the studies included, i.e., different definitions of hypothermia and different temperature measurement methods, as well as differences in patient characteristics, including sex, age, and injury severity. Accordingly, sensitivity analysis of the subgroup of studies with similar inclusion criteria regarding the ISS revealed lower heterogeneity. However, as hypothermia had a strong impact on mortality in all studies included and the subgroups assessed in sensitivity analysis, heterogeneity did not relevantly affect the finding of this meta-analysis.

Third, the effect of hypothermia on mortality may have been confounded by the higher injury severity, deranged physiology, and higher transfusion requirements in hypothermic patients. However, eleven of the studies included revealed hypothermia as an independent predictor for higher mortality, even after adjustment for the injury severity and various covariates in multivariable analysis [6, 8, 32, 35–42], including two studies that adjusted for blood product transfusions [38, 39]. Furthermore, as prehospital blood product transfusion is rare, a confounding of the effect of hypothermia on mortality by transfusion is unlikely in the current meta-analysis that included patients with temperature measurement at ED admission only.

Fourth, an estimation of the degree of hypothermia of the included patient cohorts is difficult, as only two studies reported the mean or median temperature [1, 36].

In conclusion, in the current systematic review and meta-analysis, accidental hypothermia was strongly associated with significantly higher in-hospital mortality both in trauma patients overall and patients with TBI specifically. These findings underscore the importance of measures to avoid accidental hypothermia in the prehospital care of trauma patients.

## Electronic supplementary material

Below is the link to the electronic supplementary material.

Supplementary file1 (DOCX 20 kb)
